# The *BRAF^V600E^* Mutation Is Not a Risk Factor for More Aggressive Tumor Behavior in Radiogenic and Sporadic Papillary Thyroid Carcinoma at a Young Age

**DOI:** 10.3390/cancers13236038

**Published:** 2021-11-30

**Authors:** Liudmyla Zurnadzhy, Tetiana Bogdanova, Tatiana I. Rogounovitch, Masahiro Ito, Mykola Tronko, Shunichi Yamashita, Norisato Mitsutake, Serhii Chernyshov, Sergii Masiuk, Vladimir A. Saenko

**Affiliations:** 1 State Institution “V.P. Komisarenko Institute of Endocrinology and Metabolism of the National Academy of Medical Sciences of Ukraine”, 69 Vyshgorodska Str., 04114 Kyiv, Ukraine; lzurnadzhy@gmail.com (L.Z.); tutlabogdanova1948@gmail.com (T.B.); m.tronko@dccie.kiev.ua (M.T.); serviccher@gmail.com (S.C.); 2 Department of Radiation Molecular Epidemiology, Atomic Bomb Disease Institute, Nagasaki University, 1-12-4 Sakamoto, Nagasaki 852-8523, Japan; saenko@nagasaki-u.ac.jp; 3 Department of Radiation Medical Sciences, Atomic Bomb Disease Institute, Nagasaki University, Nagasaki 852-8523, Japan; mitsu@nagasaki-u.ac.jp; 4 Nagasaki Medical Center, 2-1001-1 Kubara, Omura 856-8562, Japan; itohm@nagasaki-mc.com; 5 Fukushima Medical University, Hikarigaoka 1, Fukushima 960-1295, Japan; shun@nagasaki-u.ac.jp; 6 National Institute of Radiological Sciences, National Institutes for Quantum Science and Technology, 4-9-1 Anagawa, Chiba 263-8555, Japan; 7 State Institution “National Research Center for Radiation Medicine of the National Academy of Medical Sciences of Ukraine”, 53 Illienka Str., 04050 Kyiv, Ukraine; masja1979@gmail.com

**Keywords:** papillary thyroid carcinoma, BRAF^V600E^, Chernobyl, immunohistochemistry, VE1 antibody, Ki67 labeling index

## Abstract

**Simple Summary:**

Analysis of the groups of young Ukrainian patients (aged ≤28 years) with radiogenic and sporadic papillary thyroid carcinomas (PTCs) showed that the frequency of BRAF^V600E^ was increasing with patient age, consistently remaining lower in radiogenic PTCs. In both etiopathogenic groups, the BRAF^V600E^-positive PTCs more frequently had a dominant papillary growth pattern, smaller tumor size, higher Ki67 labeling index, and a frequency of the major indicators of tumor invasiveness that is lower than or equal to that of the BRAF^V600E^-negative tumors. Comparison of the BRAF^V600E^-positive PTCs across the groups found a virtual absence of differences, while the BRAF^V600E^-negative tumors differed markedly and displayed a higher frequency of invasive tumor features in the radiogenic PTCs. Hence, there is evidence that BRAF^V600E^ does not confer a more aggressive course of PTC in young patients regardless of tumor etiology.

**Abstract:**

Histopathological changes in the fusion oncogene-driven papillary thyroid carcinomas (PTCs) from children and adolescents exposed to Chernobyl fallout have been extensively studied. However, characteristics of the radiogenic BRAF^V600E^-positive PTCs, whose proportion is growing with time, are not well described yet. We analyzed the relationship between the BRAF^V600E^ status (determined immunohistochemically with the VE1 antibody) and the clinicopathological features of 247 radiogenic and 138 sporadic PTCs from young Ukrainian patients aged ≤28 years. The frequency of BRAF^V600E^ was increasing with patient age, consistently remaining lower in radiogenic PTCs. In both etiopathogenic groups, the BRAF^V600E^-positive PTCs more frequently had a dominant papillary growth pattern, smaller tumor size, higher Ki67 labeling index, and a frequency of the major indicators of tumor invasiveness that is lower than or equal to that of the BRAF^V600E^-negative tumors. Comparison of the BRAF^V600E^-positive PTCs across the groups found a virtual absence of differences. In contrast, the BRAF^V600E^-negative radiogenic PTCs displayed less frequent dominant papillary and more frequent solid growth patterns, lower Ki67 labeling index, and higher invasiveness than the BRAF^V600E^-negative sporadic tumors. Thus, BRAF^V600E^ is not associated with a more aggressive course of PTC in young patients regardless of etiology. The major clinicopathological differences between the radiogenic and sporadic PTCs are observed among the BRAF^V600E^-negative tumors.

## 1. Introduction

A sharp increase in the incidence of thyroid cancer among subjects aged ≤18 years in 1986 is the major health consequence of the Chernobyl accident in the exposed population. A number of studies addressing epidemiology, molecular characteristics, and clinical and pathological features of radiation-related thyroid cancer have been conducted to date.

Clinicopathological and molecular characteristics of the Chernobyl thyroid cancer, principally papillary thyroid carcinoma (PTC), have been shown to evolve with time. Tumors developing after the shorter latency (1st decade after the accident) were frequently more aggressive, especially in children and adolescents; many tumors had a solid growth pattern [1,2,3]. In contrast, PTCs diagnosed in the 2nd or 3rd decades after the accident after the longer period of latency displayed a less aggressive phenotype; the dominant papillary morphology was more common [3]. Furthermore, radiation-related PTCs in young patients were found to display pathological features of tumor aggressiveness more frequently than PTCs in non-exposed patients matched for the place of residence and age group [4].

Time-dependent changes in the clinicopathological characteristics of pediatric Chernobyl thyroid cancer were paralleled by changes in the genetic events underlying PTC. The early-onset tumors were frequently driven by fusion oncogenes such as various types of *RET/PTC*, more often *RET/PTC3* [5,6,7] followed by a shift to *RET/PTC1* after the first decade post-accident [8]. PTCs diagnosed later had a different spectrum of gene rearrangements, among which the rearranged *BRAF*, *ETV6-NTRK3* and other gene fusions were discovered [9,10,11].

Studies of point mutations in pediatric Chernobyl PTC demonstrated the absence of genetic alterations in the *RAS* family genes [10,12,13]. The mutant *BRAF* was not found in the early-onset Chernobyl PTC, then its prevalence reached approximately 10% in the mid-1990s [14] and grew to about 15% after the 2000s [9,10,15]. One of these studies claimed the frequency of mutant *BRAF* was related to patient age but not to a history of radiation exposure [15]. Of interest, the frequencies of point mutations in radiation-related PTC seems to be declining with increasing ^131^I thyroid dose, and, in contrast, an uptrend is observed for fusion genes [9,11,16]. In sporadic pediatric PTC, *BRAF^V600E^* was not detected in a small group of non-exposed children from Ukraine [15]. However, more recent works reported *BRAF^V600E^* in about one-fourth of non-exposed children and adolescents with PTC [10,17]. Each of these studies had certain strengths and limitations; the latter mostly due to the relatively small sample sizes and frequent unavailability of the appropriate control groups.

Thus, on the one hand, radiogenic PTC displays time-dependent changes in morphology and aggressiveness, and on the other, there are changes in its molecular landscape. The genotype–phenotype relationships are better understood for the tumors harboring fusion oncogenes while less is known about clinicopathological correlations for the *BRAF^V600E^*-driven PTCs in young patients.

The aim of this study was to analyze: (i) the frequency of the *BRAF^V600E^* mutation in radiogenic and sporadic PTCs from the young Ukrainian patients aged 4–28 years at diagnosis in the whole groups and age subgroups of children (aged ≤14), adolescents (aged 15–≤18) and young adults (aged ≥19 to 28); (ii) to examine the relationship of the *BRAF^V600E^* to ^131^I thyroid dose and the duration of the period of latency; and (iii) to compare the structural and invasive characteristics, proliferative activity and the frequency of postoperative recurrent lymph node metastases in the BRAF^V600E^-positive and BRAF^V600E^-negative PTCs within and across radiogenic and sporadic PTC series.

## 2. Results

### 2.1. Frequency of BRAF^V600E^ in Radiogenic and Sporadic PTC

Positive IHC reactions with the antibody to the mutant BRAF^V600E^ protein were detected in the radiogenic series in only one of 104 PTC cases (1.0%) in children, in 6/52 of cases (11.5%) in adolescents, and 19/91 of cases (20.9%) in young adults (Table 1). In contrast, BRAF^V600E^ was detected in five of 39 cases (12.8%) in children, 12/37 of cases (32.4%) in adolescents, and 25/62 cases (40.3%) in adults. In each age group, the frequency of BRAF^V600E^ in radiogenic PTC was significantly lower than in sporadic PTC. Both etiopathogenic series displayed age-related uptrends for the BRAF^V600E^ frequency (p_trend_ = 5.97 × 10^−6^ and p_trend_ = 0.004 for radiogenic and sporadic series, respectively). Logistic regression adjusted for sex also confirmed the increasing with age chance of BRAF^V600E^-positivity in radiogenic (OR = 1.179, *p* = 2.40 × 10^−5^) and sporadic (1.118, *p* = 0.007) series. Thus, the frequency of the *BRAF^V600E^* mutation in PTC is age-dependent regardless of a radiation history.

### 2.2. BRAF^V600E^ Associations in Radiogenic PTC

The results of univariate and multivariate statistical analyses are presented in Table 2. Patients with the BRAF^V600E^-positive PTC were significantly older at the time of operation than those with the BRAF^V600E^-negative tumors (b = 6.012, *p* = 3.00 × 10^−6^), and the BRAF^V600E^-positive PTCs were characterized by the longer period of latency (b = 6.133, *p* = 2.00 × 10^−6^). The latter observation is unique for the radiation-related cancers since knowledge of time points of exposure to radiation and of diagnosis enables accurate determination of latency. We found that, while a conventional proportional hazard model did not perform well (Figure S1a–c), an extended Cox model in which the BRAF status was introduced as a time-dependent variable provided reasonable fit (Figure S1d). Model parameters adequately reflected the delaying development of the BRAF^V600E^-positive PTCs (HR = 0.021, *p* = 3.67 × 10^−5^) and an increasing rate of such tumors in time (i.e., with increasing latency, HR = 1.194, *p* = 8.77 × 10^−5^ for the BRAF × Latency interaction term).

The ^131^I thyroid radiation doses in patients with BRAF^V600E^-positive PTCs were significantly lower than in patients with BRAF^V600E^-negative PTCs on univariate analysis (median 200 mGy vs. 350 mGy, respectively, *p* = 0.019) and in a multivariate model (b = −0.295, *p* = 0.031).

**Figure 1:**
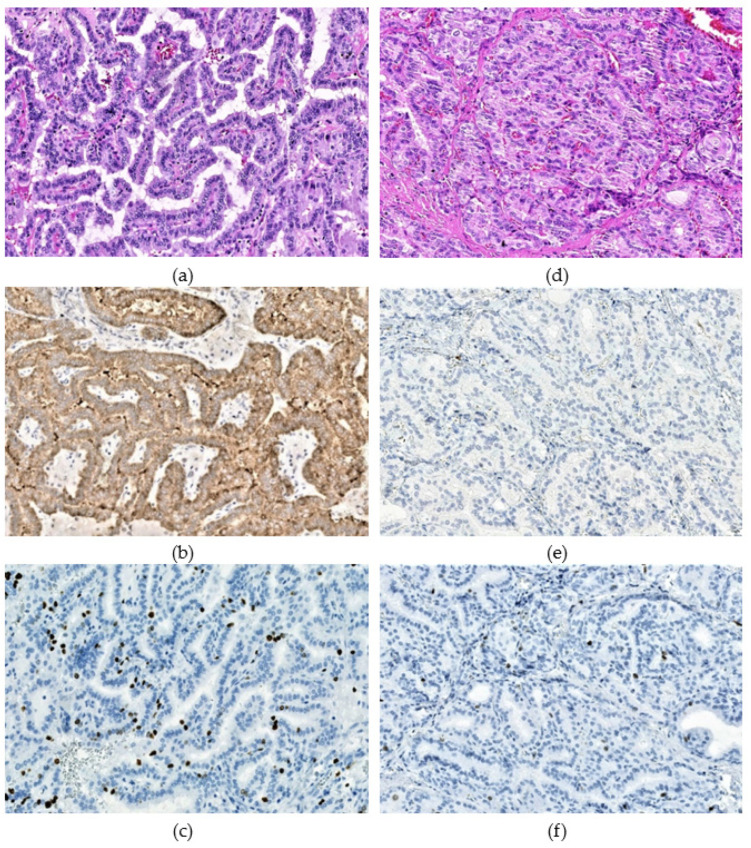
Immunohistochemical staining for BRAF^V600E^ and Ki67. (**a**–**c**) the BRAF^V600E^-positive, and (**d**–**f**) the BRAF^V600E^-negative radiogenic PTCs. (**a**)—papillary dominant growth pattern, hematoxylin-eosin, ×200; (**b**)—positive IHC reaction with the VE1 anti-BRAF (mutated V600E) antibody, ×200; (**c**)—IHC reaction with Ki67 (Clone MIB-1) antibody (Ki67 LI 8.7%), ×200; (**d**)—solid dominant growth pattern, hematoxylin-eosin, ×200; (**e**)—negative IHC reaction with the VE1 anti-BRAF (mutated V600E) antibody,×200; (**f**)—IHC reaction with Ki67 (Clone MIB-1) antibody (Ki67 LI 2.4%), ×200.

BRAF^V600E^ was statistically significantly associated with several histopathological characteristics, such as smaller tumor size ((b = −0.528, *p* = 0.013), paralleled by its higher frequency in microcarcinomas and in pT1a tumors (OR = 3.475, *p* = 0.008 for both), more frequent dominant papillary (OR = 5.735, *p* = 1.93 × 10^−4^; Figure 1a,b) and less frequent solid-trabecular dominant growth pattern than in the BRAF^V600E^-negative PTCs (OR = 0.289, *p* = 0.032; Figure 1d,e).

On multivariate analysis, BRAF^V600E^ did not confer a statistically significant association with any feature of tumor aggressiveness, including multifocality, lymphatic/vascular invasion, extrathyroidal extension, regional or distant metastasis (*p* = 0.064 for the strongest signal). For many instances, we again observed the influence of patient age at the time of operation, a powerful confounder, which rendered the effect of BRAF^V600E^ non-significant. If only sex was accounted for, a number of associations would be significant such as the lower frequencies of lymphatic/vascular invasion, extrathyroidal extension and N1b. Altogether, these observations suggest that BRAF^V600E^ is unlikely to be associated with higher tumor aggressiveness in the analyzed series of young PTC patients.

Interestingly, despite the smaller tumor size, the BRAF^V600E^ positivity was associated with the higher Ki67 LI (b = 1.674, *p* = 0.006), mostly due to a higher proportion of tumors with Ki67 LI ranging from 5% to 10% (OR = 3.974, *p* = 0.004) (Figure 1c,f)). The lower Ki67 LI (0–5%) was characterized by the significantly less frequent BRAF^V600E^ (OR = 0.268, *p* = 0.004). Ki67 LI was associated with smaller tumor size in the whole radiogenic group (b = −0.672, *p* = 0.030), but no statistical significance could be reached in the BRAF^V600E^-positive (b = −0.125, *p* = 0.848) and the BRAF^V600E^-negative (b = −0.523, *p* = 0.135) PTCs separately.

Repeated operations for recurrent PTC in regional lymph nodes performed at least 6 months after the primary surgery occurred only in the BRAF^V600E^-negative group in 8/210 (3.8%, *p* = 0.574) cases with available follow-up information. We confirmed that reoperated recurrent lymph node metastases were BRAF^V600E^-negative in all eight cases as with the primary tumors (Cohen’s κ = 1.000, *p* = 0.001). The absence of recurrences in patients with BRAF^V600E^-positive PTCs, however, did not result in statistically significant differences to the BRAF^V600E^-negative subgroup in a multivariate model (HR = 0.523, *p* = 0.708). Perhaps the small total number of recurrent events did not provide sufficient statistical power to detect the difference if any existed.

### 2.3. BRAF^V600E^ Associations in Sporadic PTC

Associations of the BRAF^V600E^-positivity with clinicopathological features in sporadic PTCs generally paralleled those in the radiogenic series (Table 3). Patients with the BRAF^V600E^-positive PTCs were significantly older at the time of operation (b = 2.516, *p* = 0.006). The BRAF^V600E^-positive tumors were smaller in size (b = −8.706, *p* = 1.52 × 10^−4^), and among those, the microcarcinomas and pT1a PTCs were more frequent (OR = 3.499, *p* = 0.003 for both). The BRAF^V600E^-positive PTCs more frequently displayed dominant papillary (OR = 5.462, *p* = 7.60 × 10^−5^) and, less frequently, follicular growth patterns (OR = 0.143, *p* = 0.001), and had more frequent oncocytic changes (OR = 2.488, *p* = 0.020).

Except for the lower frequency in tumors with lymphovascular invasion (OR = 0.304, *p* = 0.004), BRAF^V600E^ did not associate with the major indices of tumor invasiveness including multifocality, extrathyroidal extension, and regional and distant metastases (multivariate *p* = 0.068, the strongest). The integrative invasiveness score or any of its categories did not differ between the BRAF^V600E^-positive and BRAF^V600E^-negative PTCs (*p* = 0.217, the strongest).

In concordance with observations in radiogenic PTCs, sporadic BRAF^V600E^-positive tumors were associated with significantly higher proliferative activity (b = 2.221, *p* = 0.002), due to the tumors with Ki67 LI from 5 to 10% (OR = 3.109, *p* = 0.006). In the tumors with low Ki67 LI, the frequency of BRAF^V600E^ was significantly lower (OR = 0.231, *p* = 5.49 × 10^−4^) indicative of a preferential association of lower Ki67 LI with the BRAF^V600E^-negative status. Again, Ki67 LI was associated with smaller tumor size in the whole sporadic PTC group (b = −0.785, *p* = 0.005), but no statistical significance was found in the BRAF^V600E^-positive (b = −0.711, *p* = 0.056) and the BRAF^V600E^-negative (b = −0.517, *p* = 0.158) PTCs in separate analyses.

There were four recurrences in this group that were detected and reoperated six or more months after the initial surgery. Three primary tumors and reoperated metastases were BRAF^V600E^-positive, and the other and its recurring metastasis were BRAF^V600E^-negative. There was a perfect agreement in the BRAF status between the primary and recurring tumors (Cohen’s κ = 1.000, *p* = 0.001). The three BRAF-positive recurrent metastases were refractory to radioiodine treatment (not shown in the Table 3). Despite the relatively small number of patients with follow-up information in this group, the BRAF^V600E^-positivity was associated with a higher chance of recurrence on both univariate (*p* = 0.007 by the log-rank test) and multivariate analyzes (HR = 19.042, *p* = 0.031).

### 2.4. Comparison of the BRAF^V600E^-Positive or the BRAF^V600E^-Negative PTCs across the Radiogenic and Sporadic Series

Finally, we addressed the role of tumor etiology in PTCs with the same BRAF status. The BRAF^V600E^-positive PTCs from radiogenic and sporadic series were largely similar in their characteristics (Table 4). Only three parameters were statistically different: the older age at operation (b = 2.838, *p* = 0.018), lower frequency of oncocytic changes (OR = 0.242, *p* = 0.012) and longer follow-up period (b = 13.324, *p* = 8.82 × 10^−7^) in the BRAF^V600E^-positive radiogenic PTCs. Of note, there were no recurrences in the BRAF^V600E^-positive radiogenic PTCs (0/26), while 3/42 tumors recurred in the BRAF^V600E^-positive sporadic PTC subgroup. This difference was statistically significant on univariate analysis (*p* = 0.027 by the log-rank test) but not in a multivariate model (HR = 0.031, *p* = 0.147). No evidence of differences for other clinicopathological characteristics was found.

In contrast, the BRAF^V600E^-negative PTCs displayed a number of differences (Table 4). The radiogenic BRAF^V600E^-negative PTCs were more likely to be diagnosed in male patients (OR = 2.952, *p* = 0.001), more frequently displayed a solid-trabecular dominant growth pattern (OR = 2.350, *p* = 0.002), gross extrathyroidal extension (OR = 5.219, *p* = 0.008) and corresponding pT3b category (OR = 5.599, *p* = 0.006), higher invasiveness score (OR = 1.644, *p* = 0.027), and were followed-up for a longer time (b = 6.413, *p* = 3.81 × 10^−18^). Several characteristics were less frequent in the radiogenic BRAF^V600E^-negative PTCs: tumor encapsulation (OR = 0.315, *p* = 2.15 × 10^−4^), dominant papillary growth pattern (OR = 0.416, *p* = 0.002), oncocytic changes (OR = 0.298, *p* = 4.00 × 10^−5^), pT2 category (0.385, *p* = 6.49 × 10^−4^), zero invasiveness score (OR = 0.549, *p* = 0.046); the tumors also had an overall lower Ki67 LI (b = −1.714, *p* = 8.00 × 10^−6^), in particular 5–10% LI (OR = 0.488, *p* = 0.022).

**Figure 2:**
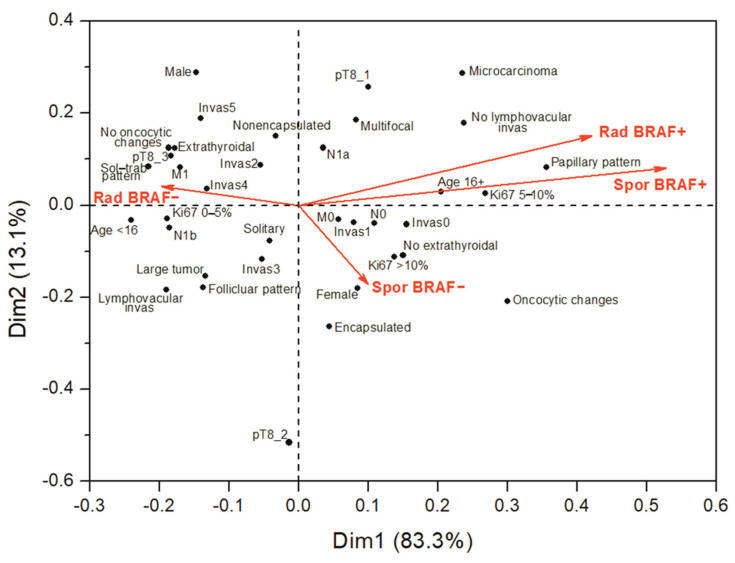
Correspondence analysis of the associations of the BRAF^V600E^-positive and BRAF^V600E^-negative PTCs of different etiology with major histopathological characteristics and tumor invasive features. The biplot displays column variables (the four PTC groups) in principal coordinates and row variables (categorical clinicopathological variables) in contribution coordinates. These coordinates present, to some extent, the association between column and row variables (e.g., the BRAF^V600E^-positive PTCs have a papillary growth pattern more frequently than the BRAF^V600E^-negative PTCs). Arrows are shown for the four PTC groups (column variables) in the graph for easier visualization of the angles between them. The smaller angle between column points (when connected to the origin) indicates the stronger correlation (e.g., the radiogenic and sporadic BRAF^V600E^-positive PTCs are rather similar), right or obtuse angle indicates the points are uncorrelated (e.g., the radiogenic BRAF^V600E^-negative PTCs are quite different from all other groups). For row points (i.e., clinicopathological characteristics), the smaller angle indicates similarity in response pattern (e.g., M0 and N0 tumors would be expected to frequently coexist, and are likely to occur in tumors with invasiveness scores 0 and 1). Dimensions 1 and 2 accounted for 96.4% of variance, and Dimension 3—for the remaining 3.6%.

The obtained results indicated that the major difference in clinicopathological characteristics between the radiogenic and sporadic PTCs were seen between the BRAF^V600E^-negative PTCs. Since the absence of statistically significant differences between the BRAF^V600E^-positive PTCs might be due in part to the relatively small sample size, thus insufficient statistical power, we performed a correspondence analysis to visualize the similarities or differences between the etiological groups of PTCs with different BRAF status. This type of statistical analysis employs mathematical and computation apparatuses different from those in the regression analysis and conceptually relates to principal component analysis but intended to explore and graphically present relationships between categorical (qualitative) variables in a low-dimension plot. The results of correspondence analysis (Figure 2) clearly demonstrated an obvious similarity between the BRAF^V600E^-positive radiogenic and sporadic PTCs (note an acute angle between these groups). These tumors were concordantly associated with older patient age, frequent micro-PTC, dominant papillary growth pattern, Ki67 LI from 5% to 10%, invasiveness score 0 or 1, and with the absence of regional (N0) and distant (M0) metastases, and of lymphovascular invasion.

The BRAF^V600E^-negative PTCs displayed marked differences in their characteristics. The radiogenic BRAF^V600E^-negative PTCs were the most distinct group of tumors associated with younger patient age, solid-trabecular growth pattern, absence of oncocytic changes, higher invasiveness scores, the presence of distant metastases and low Ki67 LI. The sporadic BRAF^V600E^-negative PTCs did not display obvious association with a particular growth pattern and appeared to be less invasive. These observations were well in line with and corroborate the results of our regression analyses presented above in Table 2, Table 3 and Table 4.

Cumulatively, our analyses suggest a clinicopathological resemblance of the BRAF^V600E^-positive radiogenic and sporadic PTCs and indicate that such tumors are not associated with the higher tumor aggressiveness in young patients.

## 3. Discussion

Here, we analyzed the clinicopathological characteristics of PTCs in the groups of young patients exposed or not to Chernobyl radiation in Ukraine with regard to the *BRAF* mutational status. The availability of two groups of patients with PTCs of different etiologies made the analyses within and across the groups possible.

First, our study confirmed the notion that the frequency of the *BRAF^V600E^* mutation in PTC increases with patient age (see Table 1). Literature data on age correlations of *BRAF^V600E^* are sometimes controversial, either confirming [15,16,18,19,20,21,22,23,24,25,26,27,28] (meta-analysis [29]) this or not [30,31,32,33] (meta-analyses [34,35]). The controversy may likely be explained by the rarity of PTC in early childhood and thus insufficient statistical power in some studies to detect age relatedness due to the underrepresentation of very young patients (i.e., lower age variability) in the groups available for analysis. Analyses of the *BRAF^V600E^* association with age in young patients from Fukushima, whose age distribution is relatively close to that in the present study, clearly indicated the increasing *BRAF* mutational frequency with patient age [24,28]. Our data also showed that the frequency of *BRAF^V600E^* in PTC rapidly increases with patients’ age transition from childhood to adolescence, and that this change is observed in both exposed and non-exposed patients. Of importance, however, the frequency of *BRAF^V600E^* remains consistently higher in sporadic PTCs as compared to radiogenic PTCs in all age subgroups. The latter observation is in line with a recent report on molecular findings in childhood PTC from Belarus [17].

To the best of our knowledge, the group of radiation-exposed children aged ≤14 years at operation for PTC presented in this study is the largest in the world (104 patients) who have been examined for the presence of *BRAF^V600E^* in the tumor. Earlier works were concordant in detecting no *BRAF^V600E^* in PTCs from exposed children of this age [10,14,15]. Our study demonstrates that *BRAF^V600E^* may exist in PTCs from such patients, albeit exceptionally rare. The BRAF^V600E^-positive radiogenic PTCs have a significantly longer period of latency (the time between exposure and tumor detection, see Table 2) resulting in the older age of patients at diagnosis. Naturally, because of the longer latency, the vast majority of children already migrate to older age groups by the time a BRAF^V600E^-positive tumor becomes detectable. In our study, the shortest period of latency of a BRAF^V600E^-positive PTC was 11.3 years; the tumor was diagnosed in a girl exposed to radiation at the age of 2.2 years. This example illustrates that only a combination of two rare factors, a very young age at exposure and a relatively short period of latency (as compared to the median of 22.1 years) for a BRAF^V600E^-positive PTC may create a condition when the patient’s age at diagnosis remains below 14 years old. Therefore, most BRAF^V600E^-positive PTCs occur in adolescents and adults in the radiogenic group. Our analysis of the BRAF^V600E^-positive and BRAF^V600E^-negative PTC onset in time after exposure also attests to the shift of the BRAF^V600E^-positive tumor development toward the longer periods of latency (see Figure S1).

In the radiogenic group, we also confirmed that the *BRAF^V600E^* mutation was associated with a lower radiation dose to the thyroid reported earlier in the Ukrainian Chernobyl PTCs [9,11,16]. Note that our study is independent of the previous works with minimal, if any, overlap in included cases.

Our analysis of clinicopathological correlations of the *BRAF^V600E^* mutation demonstrated a good concordance in the association pattern between the two etiological groups (see Table 2 and Table 3). There were a few clinicopathological characteristics’ associations which were statistically significant in one or another group, but we do not interpret those as etiology-specific since OR estimates corresponded to each other (i.e., ORS were concordantly greater or less than the value of 1). In contrast, the difference in hazard ratios for the recurrence between sporadic and radiogenic groups (HR = 19.042, *p* = 0.031 and HR = 0.523, *p* = 0.708, respectively) is suggestive that the *BRAF^V600E^* mutation may be a risk factor for recurrence at least in sporadic PTC even in young patients, which corresponds to the results of meta-analyses in adult patients [29,36,37,38,39,40].

The association of *BRAF^V600E^* with the higher Ki67 LI is of particular interest. Such a correlation was reported in adult PTC patients [18,41] but no data are available for young patients. In general, the higher Ki67 LI was associated with the greater size of PTCs in adult patients [18,42,43,44] and poorer prognosis [30,45,46,47]. The *BRAF^V600E^* mutation was also associated with the greater tumor size in some studies (meta-analyses [34,35]), although equivocally [24,27,28] (meta-analysis [39]), and with higher tumor aggressiveness (meta-analyses [29,34,35,36,38,39,48]). Our findings, therefore, provide somewhat counterintuitive yet robust evidence that, in young patients, the *BRAF^V600E^*-positive tumors, which are characterized by the higher Ki67 LI and smaller size, do not display a more aggressive phenotype. These results are in line with several previous reports of the lack of overly aggressive features in the *BRAF^V600E^*-positive PTC in pediatric patients [17,24,49,50,51,52,53].

It is tempting to speculate that, biologically, the *BRAF^V600E^*-positive PTCs with higher Ki67 LI, smaller size and diagnosed in older patients may comprise the tumors that were developing after the longer “silent” period (thus, older patient age and/or longer period of latency in exposed patients) after entering into the phase of more active growth (thus, elevated KI67 LI) when they were detected, but at this stage having not yet acquired the aggressive features observed in the *BRAF^V600E^*-positive PTCs in adult/elderly patients. In this regard, it would be interesting to investigate age-related changes of tumor aggressiveness and long-term outcomes in the *BRAF^V600E^*-positive PTCs in a special group of patients aged from children to old age. The absence of correlations between *BRAF^V600E^* and the aggressive features of PTCs in young patients does not rule out that at least some of these tumors might develop a more aggressive phenotype with time (and thus in more aged patients). Alternatively, some tumors in young patients may not be progressive, as was seen in Fukushima patients [54] in whom the frequency of the *BRAF^V600E^* mutation is about 70% [24,28]. Perhaps some *BRAF^V600E^*-positive PTCs from our series with lower Ki67 LI (about 40% of all BRAF^V600E^-positive tumors had KI67 LI ≤5%, see Table 2 and Table 3) might be those that would not progress.

Finally, we compared the BRAF^V600E^-positive and BRAF^V600E^-negative PTCs across the radiogenic and sporadic groups. Since a pronounced similarity was observed between the clinicopathological association patterns of the BRAF^V600E^-positive tumors between the radiogenic and sporadic PTC groups mentioned above, it was somewhat expected that the BRAF^V600E^-positive PTCs did not display many differences upon their comparisons in the two etiological groups (see Table 4).

In contrast, the BRAF^V600E^-negative PTCs displayed a number of differences, both in tumor morphology and the frequencies of invasive features, pointing at the higher aggressiveness of radiogenic tumors, in line with our previous report [4]. The BRAF^V600E^-negative PTCs are most likely driven by fusion oncogenes, whose histopathological associations have been established in earlier works [6,7,9,11,15,55]. Of importance, however, there are differences in the distribution of fusion oncogene types in the tumors of different etiological groups. The radiogenic PTCs, especially those detected after the shorter latency, had a very high frequency of *RET/PTC3* rearrangements, which is associated with more aggressive tumor phenotype and a solid-trabecular growth pattern [5,6,7,8,56,57]. The frequency of *RET/PTC3* is lower in sporadic PTC, even from young children, and other types of activated oncogenes do not seem to confer very high tumor aggressiveness in young patients [17,58].

Our work has several strengths and limitations. The strengths include a relatively large number of young patients in the study, the availability of two etiological groups of PTCs enabling the analyses within and across the groups, the high quality of demographic and clinicopathological information, knowledge of individual radiation doses to the thyroid, and data on the *BRAF* status, which was the focus of this investigation. The major limitation is the lack of molecular analyses for other oncogenic drivers, knowledge of which could facilitate more sophisticated and more detailed assessment of clinicopathological relationships in radiogenic and sporadic PTCs in young patients. Overcoming this shortcoming, however, is connected to several technical and practical issues, including the unavailability of tissues suitable for molecular analyses in some cases or the insufficient quality of nucleic acids from formalin-fixed paraffin-embedded tissues after a long-term storage.

## 4. Materials and Methods

### 4.1. Patients

The radiogenic cases included PTCs from 247 patients aged 6–28 years at diagnosis who were operated at the State Institution “V.P. Komisarenko Institute of Endocrinology and Metabolism of the National Academy of Medical Sciences of Ukraine” (IEM), Kyiv during the period from 1990 to 2014, that is, since the significant increase in thyroid cancer incidence after the Chernobyl accident [4,59,60]. Given that the highest thyroid cancer risk was observed in the youngest children who lived in the most contaminated by ^131^I regions of northern Ukraine [60,61], we defined radiogenic cases as those diagnosed in children aged ≤4 years in April, 1986, who lived in the Kyiv, Chernihiv and Zhytomyr regions. Additionally, the patients were divided into children (≤14), adolescents (15–18) and young adults (19–28 years old at diagnosis) for subgroup analysis.

The group of comparison, sporadic PTC cases, were from 138 patients aged 4–28 years at diagnosis born after the Chernobyl accident (from 1 January 1987 or later, i.e., not affected by ^131^I), who corresponded by age at operation and regions of residence (including Kyiv-city) to those of patients with radiogenic PTC. Patients were operated for PTC from 1997 to 2015. Since the oldest patient with sporadic PTC at the time of this study was aged 28 years, the upper limit for the age of patients with radiogenic PTC was also set to 28 years.

All cases in this work are a subset of our earlier histopathological study that included 301 radiogenic and 194 sporadic PTCs from patients aged up to 28 years [4]. Only those cases with sufficient tumor tissues remaining in the paraffin blocks were included in the current investigation to enable immunohistochemical staining.

### 4.2. Histopathology

The histopathological examination of hematoxylin/eosin-stained paraffin sections was performed by two experienced pathologists of IEM (TB and LZ). The pathological diagnosis was based on the 4th edition of the WHO histological classification [62]. Most cases were also reviewed by the international pathology panel of the CTB project [63,64]. The diagnosis of PTC was confirmed in all analyzed cases. TNM categories were determined according to the 8^th^ edition of the pTNM classification [65]. Tumors were classified according to the dominant histological growth pattern into three categories: papillary, follicular or solid-trabecular, when the corresponding structural component exceeded 50% of a tumor section surface, and was also evaluated for the presence of oncocytic (oxyphilic/Hurtle) cell changes in the tumor epithelium.

As in our previous work [60], in addition to conventional clinicopathological features, we used an integrative variable, the “invasiveness score”, which is the arithmetic sum of every instance of multifocality, lymphatic/vascular invasion, any extrathyroidal extension (i.e., minimal or gross), N1 and M1 (commonly detected by diagnostic imaging), either isolated or in combination with other(s), for each tumor. Thus defined, the invasiveness score ranged from 0 (no invasive feature presents) to 5 (all features present).

### 4.3. Immunohistochemistry

Immunohistochemical (IHC) staining for BRAF^V600E^ was performed according to the protocol used in the Department of Radiation Molecular Epidemiology of the Atomic Bomb Diseases Institute, Nagasaki University (LZ, TIR) using mouse monoclonal anti-BRAF (mutated V600E) antibody (VE1) ab228461 (Abcam, Tokyo, Japan) at a 1:100 dilution applied for one hour at 37 °C. The Novolink Polymer Detection System (250T) (Leica RE7140-K) was used to detect the IHC reaction product, which included treatment with the secondary rabbit anti-mouse antibody (IgG), attachment of the peroxidase label and visualization with DAB diluted in the Novolink DAB Substrate Buffer according to the manufacturer’s recommendations. Cell nuclei were stained with Mayer’s hematoxylin. The IHC reaction was considered positive (expression of the BRAF^V600E^ mutant protein) in the presence of the brown color of the cytoplasm of tumor epithelial cells. Sections of a formalin-fixed paraffin-embedded tumor tissue from a patient not related to this study with PTC with the *BRAF^V600E^* mutation confirmed by Sanger sequencing were used as a positive control.

We considered a positive IHC reaction for BRAF^V600E^ as indicating the presence of the *BRAF^V600E^* mutation at the DNA level. A high concordance between IHC methods employing the VE1 antibody and PCR- or direct sequencing-based techniques was confirmed in a recent meta-analysis [66], and also by our group using formalin-fixed paraffin-embedded material [67].

The proliferative activity of tumors was evaluated by IHC using Ki67 antibody (clone MIB-1; DAKO, Glostrup, Denmark, 1:100 dilution) in a Ventana BenchMark ULTRA instrument. The Ki67 LI was determined with the image-analyzing software (CountσCell, Ki67 antigen Semi Auto Counter, Seiko Tec LTD, Fukuoka, Japan) by counting Ki67-positive and -negative PTC nuclei in the tumor areas with the highest number of immunoreactive nuclei, and calculating the proportion of Ki67-positive nuclei. For each case, a total of approximately 1000 PTC cells were analyzed (LZ). Image analysis was performed in a blind for the BRAF^V600E^ status manner.

### 4.4. Thyroid Dosimetry

^131^I thyroid radiation doses were calculated for each patient from the radiogenic series in the Dosimetry department of the State Institution “National Research Center for Radiation Medicine of the National Academy of Medical Sciences of Ukraine”, Kyiv, using an ecological dosimetric model, which includes the system of ecological iodine transport and biokinetic models of iodine (“TD-CTB”) [62].

### 4.5. Statistical Analysis

The Fisher’s exact test, the Fisher–Freeman–Halton exact test and the Cochran–Armitage test were used for univariate analysis of categorical data; the Mann–Whitney test was used to compare continuous data between any two groups. Logistic regression models were adjusted for age at operation and sex; models with very small numbers of outcomes (<5 per cell) were conducted using Firth’s approach to bias-reducing penalized maximum likelihood fit. Multivariable linear regression models were used for continuous dependent variables. The occurrence of the *BRAF^V600E^* mutation in relation to the period of latency was assessed using survival analysis methods. The Kaplan–Meier method, and the proportional hazard (Cox) and extended proportional hazard models were used. Computation and plotting of the results of the model with time-varying coefficients was performed with an SAS macro “coxtvc” [68]. Calculations were performed using IBM SPSS Statistics Version 24 software (International Business Machines Corp., Armonk, NY, USA) or the 9.4 version of SAS (SAS Institute, Cary, NC, USA). Correspondence analysis was performed in R with the “ca” package [69]. The “colgreen” option was used to calculate biplot principal coordinates for the four PTC groups and contribution coordinates (the standard coordinates multiplied by the square root of the corresponding masses) for categorical clinicopathological features. All tests were two-sided; *p* < 0.05 was considered statistically significant.

## 5. Conclusions

Our study demonstrates that the *BRAF^V600E^* mutation is more frequent in sporadic than in radiogenic PTCs in all age groups of patients, and that mutational frequency significantly increases with patient age in both radiogenic and sporadic PTCs. The thyroid radiation dose in patients with the BRAF^V600E^-positive PTC was significantly lower, and the period of latency was significantly longer compared to that in patients with BRAF^V600E^-negative PTC. The major histopathological differences between the radiogenic and sporadic PTCs were observed among the BRAF^V600E^-negative tumors; the radiogenic PTCs displayed morphological features of tumor aggressiveness more frequently than the sporadic ones; the latter had a somewhat milder clinical phenotype. The BRAF^V600E^-positive PTCs displayed similar clinicopathological association patterns in the radiogenic and sporadic series, including a high frequency of microcarcinomas, dominant papillary growth pattern, high Ki67 LI, and the presence of oncocytic changes in tumor epithelial cells. The BRAF^V600E^-positive PTCs were characterized by invasive properties that were lower than or comparable to those in the BRAF^V600E^-negative tumors in both radiogenic and sporadic series, indicating that the *BRAF^V600E^* mutation is not associated with more aggressive tumor behavior in patients of young age regardless of PTC etiology. Further studies, ideally addressing all driver oncogenes and other cancer genes at the genomic and transcriptomic levels, would be desired to determine whether the phenotype and prognosis of the BRAF^V600E^-positive radiogenic PTCs will be acquiring patient age-related changes similarly to those described in sporadic PTCs.

## Figures and Tables

**Table 1:** Frequency of BRAF^V600E^ in radiogenic and sporadic PTC in different age groups at the time of operation.

Age Groups	Radiogenic PTC (*n* = 247)		Sporadic PTC (*n* = 138)		*p*-Value ^1^
	Number	%	Number	%	
Children aged ≤14 years	1/104	1.0	5/39	12.8	**0.006**
Adolescents aged 15–18 years	6/52	11.5	12/37	32.4	**0.030**
Young adults aged 19–28 years	19/91	20.9	25/62	40.3	**0.011**
Total	26/247	10.5	42/138	30.4	**2.67 × 10^−6^**
Age trend, *p*-value ^2^	**5.97 × 10^−6^**		**0.004**		
Age association ^3^	**1.179 (1.092–1.273); 2.40 × 10^−^^5^**		**1.118 (1.031–1.212); 0.007**		

^1^ The Fisher’s exact test. ^2^ The Cochran-Armitage test for trend, two-sided. ^3^ OR (95% CI) adjusted for sex; *p*-value. Numbers in bold indicate statistical significance.

**Table 2:** Characteristics of the BRAF^V600E^-positive and BRAF^V600E^-negative radiogenic PTCs.

Parameters	BRAF^V600E^(+) (*n* = 26)	BRAF^V600E^(−) (*n* = 221)	*p*-Value	OR, b or HR (95%CI)	*p*-Value
	Number or Value (% or IQR)	Number or Value (% or IQR)	Univariate	Multivariate ^1^	
**Sex (F/M, %M, F:M ratio; ref = F)**	20/6 (23.1%; 3.3:1)	141/80 (36.2%; 1.8:1)	0.276	0.706 (0.260–1.914) ^2^	0.494
**Age at operation**, years	24.1 (18.7–27.3)	15.4 (12.0–21.3)	**2.03 × 10^−6^**	**6.012 (3.552–8.472)** ^3^	**3.00 × 10^−6^**
**Age at exposure**, years	2.0 (1.0–3.0)	2.0 (1.0–3.0)	0.924	−0.019 (−0.519–0.481) ^3^	0.941
**Period of latency**, years	22.1 (17.5–24.9)	13.1 (9.3–18.5)	**1.24 × 10^−6^**	**6.133 (3.679–8.588)** ^3^	**2.00 × 10^−6^**
**Radiation dose to the thyroid, mGy**	200 (134–390)	350 (173–825)	**0.019**	**−0.295 (−0.563–0.027)** ^3^	**0.031**
**Tumor size**, mm, median	10 (6–20)	16 (12–30)	**4.57 × 10^−4^**	**−0.528 (−0.943–0.113)**	**0.013**
≤10 mm (microcarcinoma)	14 (53.8%)	36 (16.3%)	**5.44 × 10^−5^**	**3.475 (1.380–8.751)**	**0.008**
**Complete tumor capsule**	5 (19.2%)	32 (14.5%)	0.560	0.721 (0.239–2.174)	0.561
**Dominant growth pattern**			**1.98 × 10^−6^**	**0.334 (0.180–0.622)**	**0.001**
papillary	17 (65.4%)	40 (18.1%)	**1.12 × 10^−6^**	**5.735 (2.289–14.366)**	**1.93 × 10^−^^4^**
follicular	5 (19.2%)	75 (33.9%)	0.183	0.410 (0.141–1.190)	0.101
solid-trabecular	4 (15.4%)	106 (48.0%)	**0.001**	**0.289 (0.093–0.901)**	**0.032**
**Oncocytic changes**	8 (30.8%)	31 (14.0%)	**0.042**	1.671 (0.629–4.441)	0.303
**Multifocality**	6 (23.1%)	30 (13.6%)	0.235	1.124 (0.395–3.194)	0.827
**Lymphatic/vascular invasion**	9 (34.6%)	159 (71.9%)	**1.91 × 10^−4^**	0.417 (0.165–1.052)	0.064
**Extrathyroidal extension (any)**	7 (26.9%)	119 (53.8%)	**0.012**	0.565 (0.216–1.473)	0.243
**N category (N1)**	9 (34.6%)	122 (55.2%)	0.061	0.649 (0.264–1.592)	0.345
N1a	6 (23.1%)	43 (19.5%)	0.611	1.210 (0.434–3.376)	0.715
N1b	3 (11.5%)	79 (35.7%)	**0.014**	0.392 (0.109–1.406)	0.151
**M category (M1)**	1 (3.8%)	38 (17.2%)	0.091	0.340 (0.042–2.723)	0.309
**pT**			0.078	0.605 (0.310–1.176)	0.139
pT1	21 (80.8%)	126 (57.0%)	**0.021**	2.437 (0.854–6.959)	0.096
pT1a	14 (53.8%)	36 (16.3%)	**5.40 × 10^−5^**	**3.475 (1.380–8.751)**	**0.008**
pT1b	7 (26.9%)	90 (40.7%)	0.206	0.658 (0.255–1.700)	0.387
pT2	2 (7.7%)	42 (19.0%)	0.185	0.338 (0.074–1.542)	0.161
pT3	3 (11.5%)	53 (24.0%)	0.216	0.640 (0.174–2.350)	0.501
pT3a	1 (3.8%)	17 (7.7%)	0.703	0.634 (0.075–5.385)	0.676
pT3b	2 (7.7%)	36 (16.3%)	0.389	0.678 (0.144–3.201)	0.623
**Invasiveness score**	1 (0–2)	2 (1–3)	0.072	0.749 (0.531–1.056)	0.100
0	10 (38.5%)	39 (17.6%)	**0.018**	1.836 (0.737–4.575)	0.192
1	4 (15.4%)	37 (16.7%)	1.000	0.603 (0.187–1.941)	0.396
2	8 (30.8%)	60 (27.1%)	0.651	1.306 (0.511–3.339)	0.577
3	4 (15.4%)	44 (19.9%)	0.794	1.321 (0.394–4.423)	0.652
4	0	29 (13.1%)	0.052	0.148 (0.008–2.724)	0.199
5	0	12 (5.4%)	0.621	0.444 (0.017–11.596)	0.626
**Ki-67 labeling index**, median	4.7 (3.8–6.3)	*n* = 210; 2.3 (1.3–3.9)	**7.64 × 10^−6^**	**1.647 (0.467–2.827)**	**0.006**
0–5%	13 (50.0%)	173 (78.3%)	**0.003**	**0.268 (0.109–0.659)**	**0.004**
>5–10%	11 (42.3%)	29 (23.1%)	**7.00 × 10^−4^**	**3.974 (1.565–10.095)**	**0.004**
>10%	2 (7.7%)	8 (3.6%)	0.284	1.351 (0.252–7.253)	0.726
**Follow-up**, years	9.9 (8.3–12.2)	13.5 (8.5–18.7)	**0.016**	0.469 (−1.978–2.916)	0.706
**LN recurrence (reoperated after 6 mo)**	0	*n* = 210; 8 (3.8%)	0.574 ^4^	0.523 (0.004–5.410) ^5^	0.708

^1^ Adjusted for age at operation and sex unless otherwise specified; characteristics of sporadic PTCs were used as references; OR from logistic regression, b-coefficient from linear regression, HR from proportional hazard (Cox) regression. ^2^ Adjusted for age at operation. ^3^ Adjusted for sex. ^4^ The log-rank test. ^5^ The Firth’s penalized proportional hazard model. Numbers in bold indicate statistical significance.

**Table 3:** Characteristics of the BRAF^V600E^-positive and BRAF^V600 E^-negative sporadic PTCs.

Parameters	BRAF^V600E^(+) (*n* = 42)	BRAF^V600E^(−) (*n* = 96)	*p*-Value	OR, b or HR (95%CI)	*p*-Value
	Number or Value (% or IQR)	Number or Value (% or IQR)^1^	Univariate	Multivariate ^1^	
**Sex (F/M, %M, F:M ratio; ref = F)**	34/8 (19.0%; 4.3:1)	81/15 (15.6%; 5.4:1)	0.626	1.378 (0.518–3.670) ^2^	0.521
**Age at operation**, years	21.0 (16.4–24.3)	17.1 (14.0–21.7)	**0.006**	**2.516 (0.748–4.284)** ^3^	**0.006**
**Tumor size**, mm	11 (8–15)	21 (13–31)	**2.64 × 10^−5^**	**−8.706 (−13.122–4.290)**	**1.52 × 10^−4^**
≤10 mm (microcarcinoma)	19 (45.2%)	18 (18.8%)	**0.002**	**3.499 (1.531–7.966)**	**0.003**
**Complete tumor capsule**	6 (14.3%)	32 (33.3%)	**0.023**	**0.231 (0.083–0.648)**	**0.005**
**Dominant growth pattern**			**1.72 × 10^−4^**	**0.427 (0.248–0.736)**	**0.002**
papillary	31 (73.8%)	35 (36.5%)	**7.91 × 10^−5^**	**5.462 (2.355–12.666)**	**7.60 × 10^−5^**
follicular	4 (9.5%)	33 (34.4%)	**0.003**	**0.143 (0.045–0.460)**	**0.001**
solid-trabecular	7 (16.7%)	28 (29.2%)	0.141	0.565 (0.219–1.460)	0.238
**Oncocytic changes**	26 (61.9%)	36 (37.5%)	**0.010**	**2.488 (1.155–5.358)**	**0.020**
**Multifocality**	10 (23.8%)	11 (11.5%)	0.075	1.905 (0.712–5.095)	0.199
**Lymphatic/vascular invasion**	13 (31.0%)	62 (64.6%)	**7.46 × 10^−6^**	**0.304 (0.134–0.688)**	**0.004**
**Extrathyroidal extension (any)**	11 (26.2%)	33 (34.4%)	0.543	0.897 (0.382–2.106)	0.803
**N category (N1)**	15 (35.7%)	42 (43.8%)	0.454	0.793 (0.362–1.739)	0.562
N1a	11 (26.2%)	16 (16.7%)	0.244	1.696 (0.682–4.214)	0.255
N1b	4 (9.5%)	26 (27.1%)	**0.025**	0.343 (0.109–1.084)	0.068
**M category (M1)**	1 (2.4%)	5 (5.2%)	0.407	0.445 (0.049–3.998)	0.469
**pT**			0.078	**0.259 (0.115–0.580)**	**0.001**
pT1	35 (83.3%)	48 (50.0%)	**2.72 × 10^−4^**	**5.098 (2.017–12.883)**	**0.001**
pT1a	19 (45.2%)	18 (18.8%)	**0.002**	**3.499 (1.531–7.996)**	**0.003**
pT1b	16 (38.1%)	30 (31.3%)	0.440	1.427 (0.651–3.130)	0.374
pT2	6 (14.3%)	36 (37.5%)	**0.008**	**0.269 (0.101–0.719)**	**0.009**
pT3	1 (2.4%)	12 (12.5%)	0.109	0.175 (0.021–1.422)	0.103
pT3a	1 (2.4%)	9 (9.4%)	0.282	0.227 (0.027–1.904)	0.172
pT3b	0	3 (3.1%)	0.553	0.336 (0.009–12.891)	0.558
**Invasiveness score**	1 (0–2)	2 (0–3)	0.424	0.835 (0.607–1.147)	0.266
0	15 (35.7%)	27 (28.1%)	0.423	1.215 (0.543–2.719)	0.635
1	13 (31.0%)	20 (20.8%)	0.203	1.601 (0.688–3.728)	0.275
2	7 (16.7%)	21 (21.9%)	0.646	0.705 (0.267–1.866)	0.482
3	5 (11.9%)	21 (21.9%)	0.237	0.506 (0.171–1.491)	0.217
4	2 (4.8%)	7 (7.3%)	0.722	1.175 (0.205–6.750)	0.856
5	0	0	ND	ND	ND
**Ki-67 labeling index**	*n* = 40; 5.9 (4.3–8.7)	4.1 (2.5–6.9)	**0.003**	**2.221 (0.842–3.600)**	**0.002**
0–5%	14 (35.0%)	62 (64.6%)	**0.002**	**0.231 (0.101–0.531)**	**5.49 × 10^−4^**
>5–10%	20 (50.0%)	25 (26.0%)	**0.009**	**3.109 (1.395–6.930)**	**0.006**
>10%	6 (15.0%)	9 (9.4%)	0.374	2.322 (0.716–7.534)	0.161
**Follow-up**, years	4.9 (2.4–8.6)	5.5 (2.6–9.3)	0.343	−0.144 (−1.787–1.499)	0.863
**LN recurrence (reoperated after 6 mo)**	*n* = 39; 3 (7.7%)	*n* = 88; 1 (1.1%)	**0.007** ^4^	**19.042 (1.299–279.067)** ^5^	**0.031**

^1^ Adjusted for age at operation and sex unless otherwise specified; characteristics of the sporadic PTCs were used as references; OR from logistic regression, b-coefficient from linear regression, HR from proportional hazard (Cox) regression. ^2^ Adjusted for age at operation. ^3^ Adjusted for sex. ^4^ The log-rank test. ^5^ The proportional hazard model. Numbers in bold indicate statistical significance.

**Table 4:** Statistical comparison of the BRAF^V600E^-positive or BRAF^V600E^-negative PTCs across the radiogenic and sporadic.

Characteristics	BRAFV600E(+) (*n* = 26/42) ^1^			BRAFV600E(−) (*n* = 221/96) ^1^		
	Univariate	OR, b or HR (95%CI) ^2^	Multivariate	Univariate	OR, b or HR (95%CI) ^2^	Multivariate
	*p*-Value		*p*-Value	*p*-Value		*p*-Value
**Sex** (ref = F)	0.762	1.357 (0.389–4.731) ^3^	0.632	**1.80 × 10^−4^**	**2.952 (1.590–5.482)** ^3^	**0.001**
Age at operation, years	**0.015**	**2.838 (0.496–5.180)** ^4^	**0.018**	0.070	−0.593 (−2.015–0.829) ^4^	0.412
**Tumor size**, mm	0.519	0.264 (−4.730–5.258)	0.916	0.224	−0.865 (−4.149–2.419)	0.605
≤10 mm (microcarcinoma)	0.619	1.262 (0.447–3.561)	0.661	0.627	0.781 (0.404–1.508)	0.461
**Complete tumor capsule**	0.737	1.516 (0.383–5.998)	0.553	**2.22 × 10^−4^**	**0.315 (0.171–0.581)**	**2.15 × 10^−4^**
**Dominant growth pattern**	0.539	0.955 (0.303–3.004)	0.937	**5.88 × 10^−4^**	**2.340 (1.472–3.721)**	**3.25 × 10^−4^**
papillary	0.585	0.938 (0.290–3.034)	0.914	**0.001**	**0.416 (0.239–0.724)**	**0.002**
follicular	0.287	1.779 (0.388–8.147)	0.458	1	0.882 (0.522–1.493)	0.641
solid-trabecular	1	0.622 (0.140–2.755)	0.531	**0.002**	**2.350 (1.370–4.032)**	**0.002**
**Oncocytic changes**	**0.024**	**0.242 (0.080–0.733)**	**0.012**	**9.00 × 10^−6^**	**0.298 (0.167–0.531)**	**4.00 × 10^−5^**
**Multifocality**	1	0.612 (0.170–2.209)	0.454	0.717	1.281 (0.600–2.737)	0.523
**Lymphatic/vascular invasion**	0.794	1.915 (0.594–6.172)	0.277	0.231	1.364 (0.787–2.366)	0.269
**Extrathyroidal extension**	1	0.946 (0.294–3.043)	0.925	**0.001**	**2.115 (1.244–3.595)**	**0.006**
minimal	1	0.804 (0.242–2.667)	0.721	0.253	1.255 (0.737–2.138)	0.403
gross	0.382	3.224 (0.137–60.094)	0.433	**0.001**	**5.219 (1.532–17.781)**	**0.008**
**N category (N1)**	1	0.791 (0.264–2.372)	0.675	0.067	1.438 (0.871–2.374)	0.155
N1a	1	0.683 (0.201–2.321)	0.541	0.639	1.187 (0.623–2.265)	0.602
N1b	1	1.198 (0.229–6.262)	0.831	0.154	1.327 (0.765–2.302)	0.315
**M category (M1)**	1	2.504 (0.128–49.133)	0.545	0.055	2.139 (0.929–4.924)	0.074
**pT**	0.306	1.462 (0.389–5.503)	0.574	**0.001**	0.976 (0.608–1.567)	0.920
pT1	1	0.770 (0.200–2.957)	0.703	0.270	1.361 (0.830–2.231)	0.222
pT1a	0.619	1.262 (0.447–3.581)	0.661	0.627	0.781 (0.404–1.508)	0.461
pT1b	0.433	0.649 (0.214–1.974)	0.447	0.131	1.555 (0.923–2.621)	0.097
pT2	0.701	0.563 (0.099–3.211)	0.517	**6.39 × 10^−4^**	**0.385 (0.223–0.666)**	**6.49 × 10^−4^**
pT3	0.152	5.074 (0.465–55.415)	0.183	**0.023**	**2.155 (1.072–4.335)**	**0.031**
pT3a	1	2.504 (0.128–49.133)	0.545	0.658	0.842 (0.354–2.006)	0.698
pT3b	0.143	1.476 (0.036–60.523)	0.837	**6.30 × 10^−4^**	**5.599 (1.651–18.985)**	**0.006**
**Invasiveness score (any Ex)**	0.401	1.063 (0.422–2.678)	0.897	**0.024**	**1.644 (1.059–2.552)**	**0.027**
0	1	1.105 (0.379–3.222)	0.854	**0.050**	**0.549 (0.305–0.990)**	**0.046**
1	0.249	0.482 (0.133–1.754)	0.268	0.427	0.816 (0.436–1.526)	0.524
2	0.231	1.940 (0.574–6.558)	0.286	0.401	1.378 (0.770–2.464)	0.28
3	0.723	1.036 (0.228–4.714)	0.964	0.762	0.713 (0.384–1.323)	0.283
4	0.521	0.495 (0.027–8.990)	0.634	0.177	1.918 (0.792–4.644)	0.149
5	ND ^5^	ND	ND	0.021	10.119 (0.618–165.760)	0.105
**Ki-67 labeling index**	0.112 ^6^	−1.114 (−3.135–0.907)	0.275	**3.84 × 10^−9 7^**	**−1.714 (−2.455–0.973)**	**8.00 × 10^−6^**
0–5%	0.306	2.057 (0.702–6.031)	0.189	**0.001**	**2.283 (1.304–3.995)**	**0.004**
>5–10%	0.618	0.560 (0.191–1.645)	0.292	**0.015**	**0.488 (0.264–0.903)**	**0.022**
>10%	0.464	0.710 (0.117–4.312)	0.71	0.061	0.473 (0.174–1.283)	0.142
**Follow-up**, years (*n* = 26/39)	**3.39 × 10^−5^**	**13.324 (8.434–18.213)**	**8.82 × 10^−7^**	**1.50 × 10^−14^**	**6.413 (5.048–7.777)**	**3.81 × 10^−18^**
**LN recurrences (reoperated after 6 mo)**	**0.027** ^8^	0.031 (0.000–0.870) ^9^	0.147	0.872 ^8^	0.694 (0.074–6.535) ^10^	0.750

^1^ The number of radiogenic/sporadic cases. ^2^ Adjusted for age at operation and sex unless otherwise specified; characteristics of the sporadic PTCs were used as references; OR from logistic regression, b-coefficient from linear regression, HR from proportional hazard (Cox) regression. ^3^ Adjusted for age at operation. ^4^ Adjusted for sex. ^5^ Not determined. ^6^ The number of cases is 26/40. ^7^ The number of cases is 210/96. ^8^ The log-rank test. ^9^ The Firth’s penalized proportional hazard model. ^10^ The proportional hazard model. Numbers in bold indicate statistical significance.

## Data Availability

All data generated or analyzed during this study are included in this published article.

## Supplementary Materials

The following are available online at https://www.mdpi.com/article/10.3390/cancers13236038/s1, Figure S1: Checking the proportional hazard assumption for the BRAF status variable in a proportional hazard and in an extended Cox model of PTC onset in time after exposure (Latency, years) for the radiogenic PTCs.
