# Supplementary material for: The BRAFV600E Mutation Is Not a Risk Factor for More Aggressive Tumor Behavior in Radiogenic and Sporadic Papillary Thyroid Carcinoma at a Young Age
Source: Cancers (Basel). 2021 Nov 30;13(23):6038. doi: 10.3390/cancers13236038 (PMC8656579; doi:10.3390/cancers13236038)
Supplement: Supplementary file 1 [file cancers-13-06038-s001.zip › cancers-1455615-supplementary.pdf]

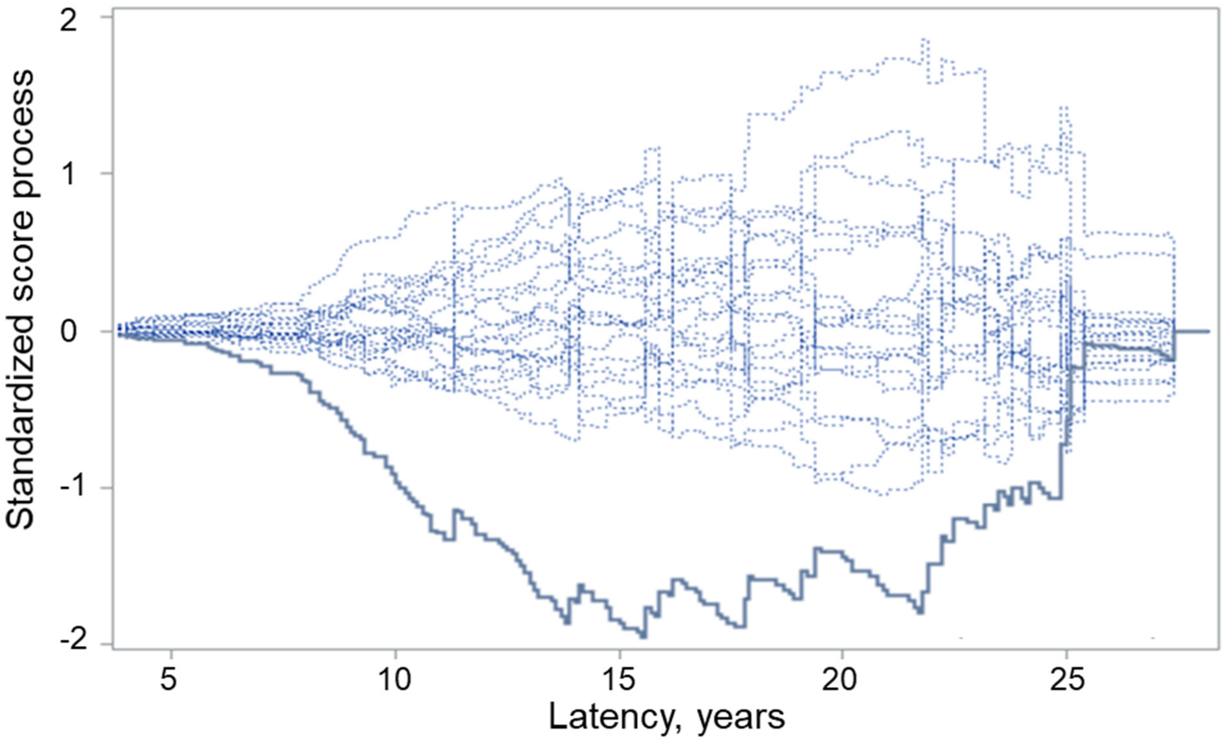

Standardized score process
2
1
0
-1
-2
5
10
15
20
25
Latency, years

a

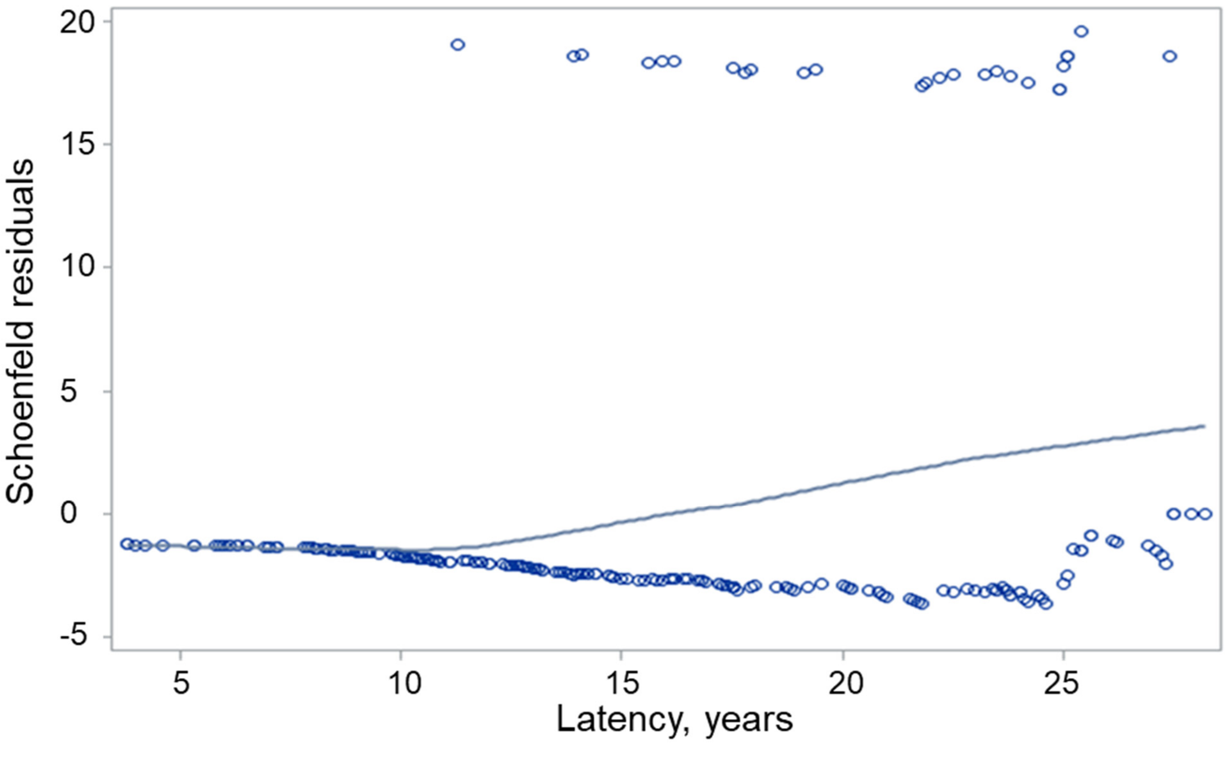

Schoenfeld residuals
20
15
10
5
0
-5
5
10
15
20
25
Latency, years

b

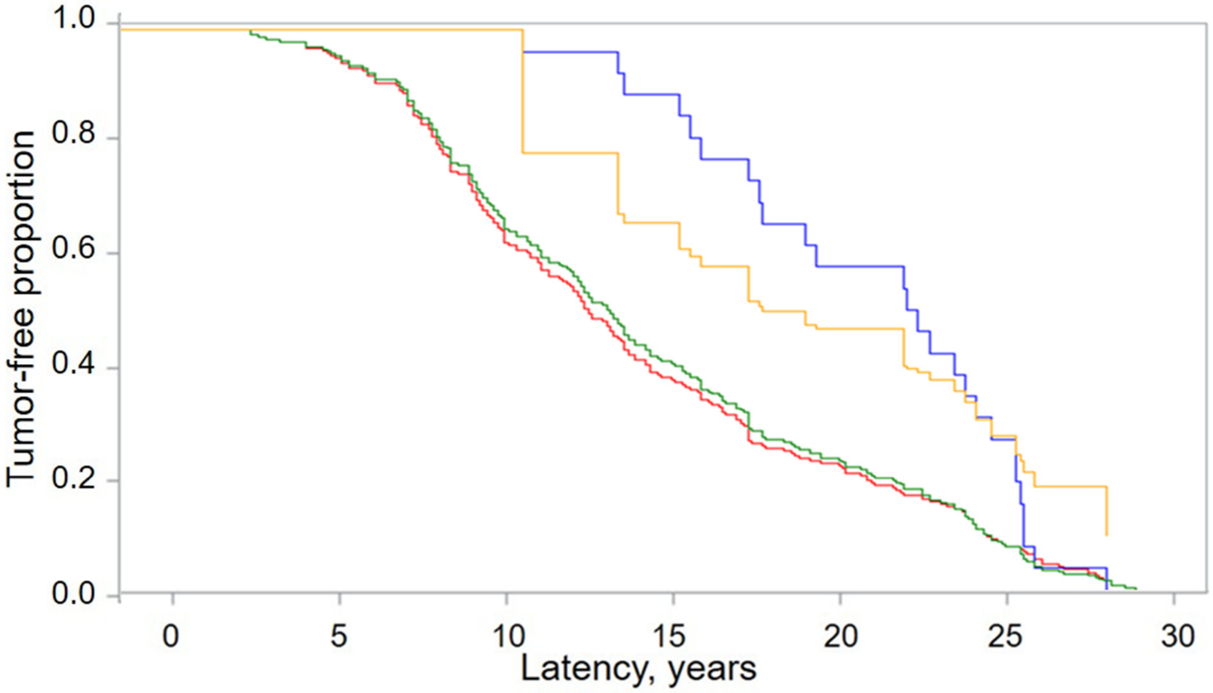

c

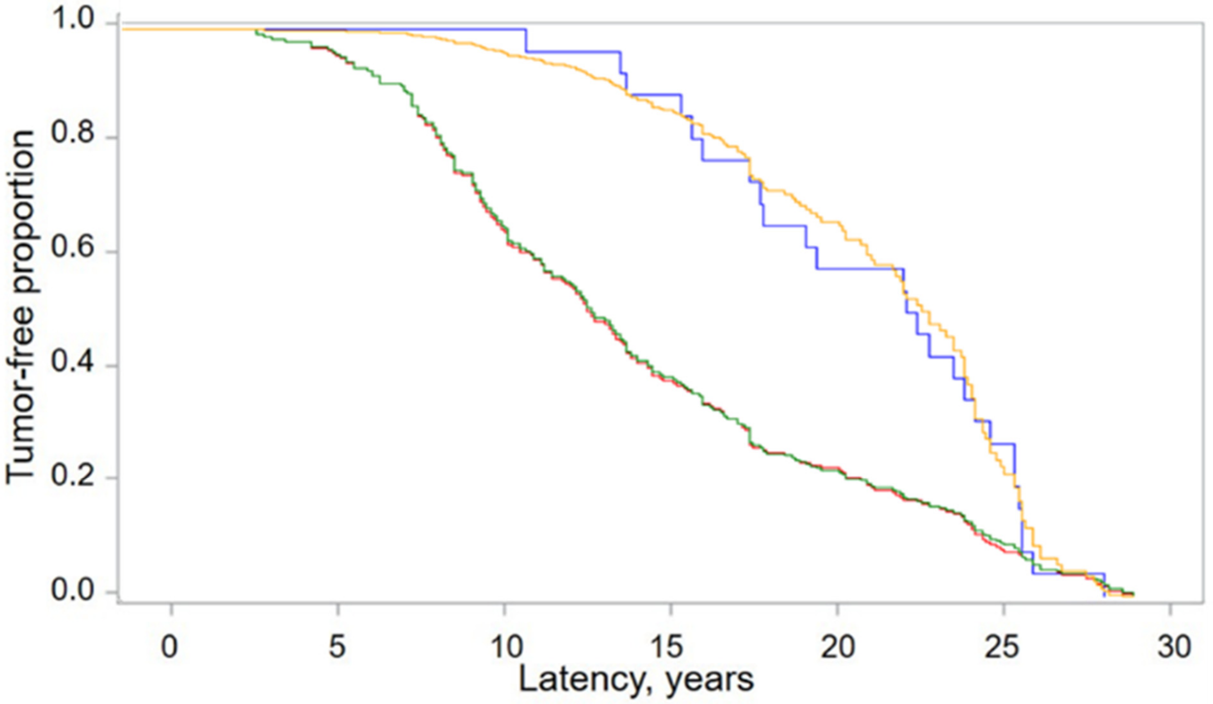

d

Figure S1. Checking the proportional hazard assumption for the BRAF status variable in a proportional hazard and in an extended Cox model of PTC onset in time after exposure (Latency, years) for the radiogenic PTCs. (**a**) – the observed standardized score process (solid line) and first 20 simulated realizations from the null distribution (dotted lines) for the BRAF status; a total of 1000 simulated realizations were tested (p=0.002 by the Kolmogorov-type supremum test); the deviation of the observed process from the pattern seen for simulated realizations is a strong indicator of nonproportional hazards for the BRAF status; diagnostics of the model for was performed using the "assess" statement with the "ph" option in PROC PHREG of SAS. (**b**) – the Schoenfeld residuals for the BRAF status variable plotted against the duration of the period of latency (circles) and a smoothing spline (smooth = 0.5, solid line); the "non-horizontal" shape of the

smoothed curve is a strong indicator of nonproportional hazards for the BRAF status; the "ressch" option in the "output out" statement in PROC PHREG of SAS were used to save the Schoenfeld residuals. (**c**) – overlaid Kaplan-Meier survival functions (blue – the $BRAF^{V600E}$-positive, red – the $BRAF^{V600E}$-negative PTCs; p= *p*=0.002 by the log-rank test) and the BRAF status (orange – the $BRAF^{V600E}$-positive, green – the $BRAF^{V600E}$-negative) survival estimates from the proportional hazard model; the model performance was poor for the $BRAF^{V600E}$-positive PTCs. (**d**) - overlaid Kaplan-Meier survival functions (blue – the $BRAF^{V600E}$-positive, red – the $BRAF^{V600E}$-negative PTCs) and the BRAF status (orange – the $BRAF^{V600E}$-positive, green – the $BRAF^{V600E}$-negative) survival estimates from the extended Cox model; the model performance was poor for the $BRAF^{V600E}$-positive PTCs.The Kaplan-Meier plots shown in (c, d) demonstrate a clear lag in the development of $BRAF^{V600E}$-positive PTCs as compared to $BRAF^{V600E}$-negative tumors for the initial 15 years after radiation exposure. For the latency from 15 to 20 years, survival functions look proportional for the two subgroups. After that, an accelerated failure time behavior of the $BRAF^{V600E}$-positive PTCs is observed. These observations were suggestive of violation of the proportional hazard assumption, which was confirmed using model diagnostics (a, b). Due to this violation, the proportional hazard model (c) did not perform well, while the extended Cox model (in which the BRAF status was introduced as a time-dependent variable) did (d).
